# Development of Micro Laser Powder Bed Fusion for Additive Manufacturing of Inconel 718

**DOI:** 10.3390/ma15155231

**Published:** 2022-07-28

**Authors:** Saeed Khademzadeh, Claudio Gennari, Andrea Zanovello, Mattia Franceschi, Alberto Campagnolo, Katya Brunelli

**Affiliations:** Industrial Engineering Department, University of Padova, 35121 Padova, Italy; saeed.khademzadeh@unipd.it (S.K.); claudio.gennari@unipd.it (C.G.); andrea.zanovello.1@studenti.unipd.it (A.Z.); mattia.franceschi@phd.unipd.it (M.F.); alberto.campagnolo@unipd.it (A.C.)

**Keywords:** Ni-based superalloys, micro laser powder bed fusion, heat treatment, mechanical properties

## Abstract

The development of laser powder bed fusion (LPBF) additive manufacturing techniques for microfabrication raises the need for the employment of new process configurations and parameters. In this study, micro-LPBF of Ni-based superalloy Inconel 718 using a spot laser of 30 µm was examined. The response surface method with a central composite design was employed to determine the optimum process parameter. A wide range of heat treatment cycles was applied to additively manufacture Inconel samples. The mechanical behavior of heat-treated Inconel 718 parts fabricated via micro-LPBF was investigated and correlated to the microstructural characteristics. The result showed that using optimum input energy density led to a homogenous distribution of nanosized (<10 nm) circular γ′ and plate-like γ″ particles in the γ matrix. Uniaxial tensile tests on heat-treated samples showed that ageing temperature is the most determinant factor in the mechanical strength of additively manufactured Inconel 718.

## 1. Introduction

Inconel 718 (Inc718) is the most widely used nickel-based superalloy [1] due to its high strength, creep, and fatigue resistance at high temperatures (up to 700 °C). Excellent resistance to oxidation and hot corrosion makes Inc718 an alloy of choice for many applications such as the aerospace industry [2]. In particular, Inc718 is used for extreme operating conditions such as gas turbines, turbochargers, nuclear reactors, liquid propellant rockets, combustors, heat exchangers, turbine blades, high-pressure vessels, and cryogenic storage tanks [3]. Due to rapid advances in technology, fabricated components are required to have increasingly complex part geometries (e.g., cooling channels in turbine blades). However, high hardness and low thermal conductivity make Inc718 a difficult to cut material because of tool over-wear and poor workpiece surface integrity after conventional machining processes [4]. Additive Manufacturing (AM) is one of the main emerging advanced manufacturing methods and has demonstrated vast application opportunities. AM technologies are capable of making complex functional parts that cannot be obtained using conventional techniques [5,6]. One of the main laser-based AM processes is laser powder bed fusion (LPBF) which is based on the melting of certain areas of the metal powder layers using a high-intensity laser, following the indications of computer-aided designed models (CAD data) [7]. The LPBF appears to be one of the most promising metal AM techniques [8] for the fabrication of geometrically complex components with high dimensional accuracy and good surface integrity [9]. A vast number of research activities have been carried out on LPBF of engineering metallic materials such as titanium and nickel alloys [10]. Therefore, LPBF as a tool-free production method was found to be an excellent candidate for the fabrication of complex geometries from Nickel-based superalloys. Inc718 parts require heat treatments to improve mechanical properties by precipitation hardening, regardless of the process used for fabrication. Generally, these treatments consist of solubilization followed by age hardening to induce the precipitation of reinforcement phases (γ′ and γ″ phases) [11].

Selecting the correct heat treatment parameters is necessary to achieve proper changes in the microstructure and mechanical properties. Chlebus et al. [12] found that solution annealing of Inc718 produced by laser powder bed fusion requires a higher temperature than that typically involved (~1100 °C instead of ~1000 °C). Huang et al. [13] studied the influence of solution time, temperature, and cooling rate as well as age hardening on the microstructure and mechanical properties of LPBF Inc718. In order to increase the productivity of the LPBF process, a laser spot greater than 50 µm is generally used for the LPBF of Inc718 [14,15]. For precision applications, on the other hand, a reduced spot size allows for producing parts with a higher resolution, recreating their features with high dimensional accuracy [16]. Recently, there has been an ever-increasing demand for micro-fabrication technologies to meet the push toward miniaturization that is taking place in various sectors [17]. Compared with other techniques commonly used for micro-AM, micro-LPBF is interesting due to several factors such as faster cycle time, material versatility, and process simplicity [18]. In the present study, several heat treatments were performed on Inc718 samples produced by micro-LPBF using a combination of optimized process parameters. Aiming to develop LPBF for micro-fabrication, a 30 µm laser spot was employed which led to a change in input energy densities and microstructural characteristics.

## 2. Materials and Methods

### 2.1. Starting Powder

**Figure 1:**
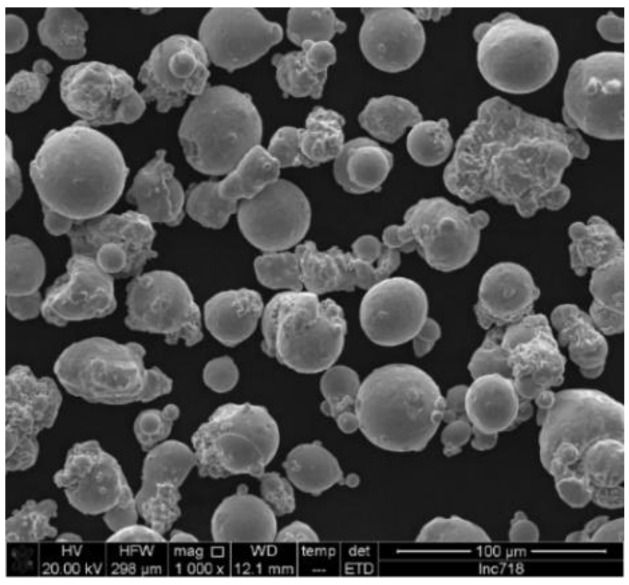
SEM micrograph of Inc718 powder produced by gas atomization.

Gas-atomized Inc718 powder produced by Böhler was used as starting material. Powder particles ranged from 15 to 45 µm. The chemical composition of the initial powder is presented in Table 1. A scanning electron microscope (SEM) showed a spherical overall shape for powder particles and the presence of irregular satellites (Figure 1). The flowability of the powder is a crucial factor in powder bed additive manufacturing technologies such as LPBF. The rheology of the powders was measured by FT4 Powder Rheometer (Freeman Technology, Tewkesbury, UK). This experiment revealed that the basic flowability energy (BFE) of the Inconel 718 powder was 940 mJ that provided a flawless layer of powder after recoating.

### 2.2. LPBF Process Set Up for Microfabrication

LPBF machine of SISMA (MYSINT100TM) was used for the fabrication of Inc718 samples. The machine is equipped with a 200 W fiber laser. A galvo scanner system consisting of quartz F-Theta Lens provides the planar movement of the laser beam. To enhance the resolution of the process, laser spot diameter was set at 30 µm which enables microfabrication via LPBF. The machine uses an x-lip rubber-like blade to spread the powder particles on the building platform. A constant argon flow over the building platform was used to remove unwanted melting products such as burnt or oxidized particles. The whole process was carried out in an argon atmosphere with an oxygen level below 500 ppm to minimize unwanted chemical reactions. The layer thickness was kept constant at 20 µm in all experiments. Support generation and slicing of computer-aided designed (CAD) models were performed using the software Magics^TM^ (Materialise, SISMA, Leuven, Belgium).

### 2.3. Attaining Process Parameters

**Figure 2:**
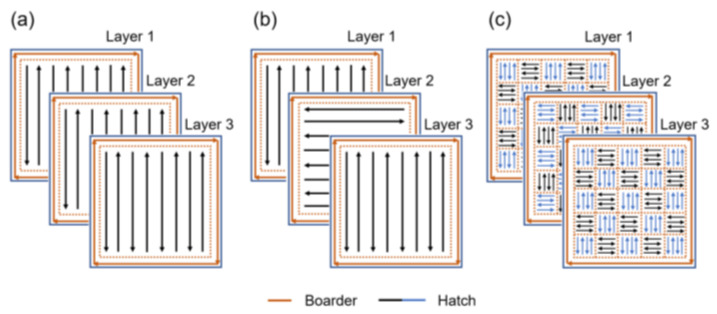
Different LPBF scanning strategies used for processing of Inc718; (**a**) bidirectional, (**b**) alternating bidirectional, (**c**) alternating chessboard.

In this study, a comprehensive design of experiment (DOE) was employed to evaluate the density and surface topography of LPBF parts corresponding to the interaction among the main LPBF process parameters such as laser power, scanning speed, hatch distance, and scanning strategy. Dense cubic (10 mm × 10 mm × 10 mm) samples were built for DOE. Input ranges of process parameters for three numeric factors and one categorical factor are summarized in Table 2. The Design–Expert (Stat-Ease Inc., Minneapolis, MN, USA) software was employed to implement a two-step DOE using response surface methodology (RSM) and subsequent full-factorial technique. A central composite design (CCD) was used which is the most widely used RSM design for the experiment. CCD efficiently locates the sampling points to increase the accuracy of the response surfaces after experimental measurement of parameters for sampling points [19]. Each numeric factor was set to five levels: high and low levels (factorial points), plus and minus alpha (axial points), and the central point. For each categorical factor, the central composite design was duplicated for every combination of the categorical factor levels. Bidirectional (Figure 2a) and alternating bidirectional (Figure 2b) scanning strategies were employed for the fabrication of CCD samples.

### 2.4. Heat Treatment Cycles

**Figure 3:**
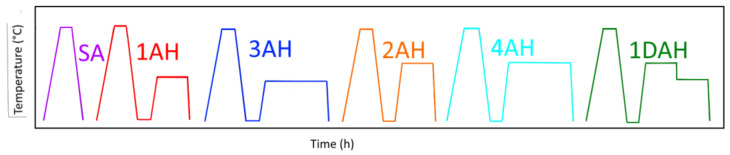
Schematic representation of 6 heat treatment cycles performed in this research.

Based on existing standard heat treatments for wrought and casting Inc718 (see Table 3), a systematic heat treatment study was performed through different heat treatment cycles performed on Inc718 cubic samples produced by optimized LPBF process parameters. These consist of a Solution Annealing (SA) followed by single Age Hardening (AH) or Double Age Hardening (DAH) at two levels of ageing temperature (620 °C, 720 °C) for 4 h and 8 h. The furnace cooling rate from the solution annealing temperature to the ageing temperature was set at 100 °C/h. The same cooling rate was applied for double ageing. Heat treatment cycles are summarized in Table 4 and schematically presented in Figure 3. All heat treatments were carried out in a CWF laboratory chamber furnace (Carbolite^®^, Derbyshire, UK) at a heating rate of 10 °C/min, in argon atmosphere.

### 2.5. Characterization of LPBF Products

#### 2.5.1. Density Measurement

Archimedes’ principle was employed to determine the density of LPBF processed Inc718. A precision balance with an accuracy of 0.1 mg was used to measure the mass of produced samples in the air (*m_air_*) and while submerged in de-ionized water (*m_w_*). Both measurements were performed five times and the arithmetic mean values were applied in Equation (1) to calculate the density:

(1) $$\rho_{s}=\frac{m_{air}}{m_{air}-m_{w}}\;\left(\rho_{w}-\rho_{air}\right)+\rho_{air}$$

where $\rho_{s}$ is the density of the as-built sample, $\rho_{w}$ is the density of de-ionized water and $\rho_{air}$ is the density of the air. Density values were reported relative to the theoretical bulk density of Inc718 ($\rho_{Bulk}$ = 8400 kg/m^3^).

#### 2.5.2. Surface Roughness Measurement

The surface roughness of the printed Inc718 samples was measured using 3D optical profilometry. In this work, a Sensofar SNeox optical 3D profiler was used in focus variation mode with a 20× objective lens (NA 0.45, field of view 877 × 660 µm, spatial sampling equal to 0.65 µm and optical resolution 0.31 µm). For each sample, a surface with an area equal to 3.68 × 3.29 mm was scanned and extracted to compute the surface parameter *Sa* which is the extension of *Ra* (arithmetical mean height of a line) to a surface.

#### 2.5.3. Microstructural Analysis

The cross-sections of cubic specimens in the building direction were prepared by wet grinding using abrasive papers (400–4000 grit) and by subsequent polishing with 1 µm diamond suspension. To reveal the microstructural features, as-built specimens were electrolytically etched using a mixture of 70 mL H_3_PO_4_ and 30 mL H_2_O at 5 V for 10 s. The heat-treated samples were instead etched with Kalling’s etchant (100 mL HCl, 100 mL ethanol, 5 g CuCl_2_). The microstructure was examined using a Leica DMRE optical microscope (OM, Leica Microsystems S.r.l., Milan, Italy) and a Leica/Cambridge Leo Stereoscan S-440 scanning electron microscope (SEM, Leica Microsystems S.r.l., Milan, Italy), and Zeiss Sigma HD field-emission SEM (FESEM) (Carl Zeiss S.p.A., Milan, Italy). Moreover, a JEOL 200CX (Jeol Ltd., Tokyo, Japan) transmission electron microscope (TEM) was employed to investigate the precipitation hardening after heat treatment cycles. For this purpose, thin foils were obtained from the samples and mechanically thinned down to approximately 50 µm in thickness. Then, 3 mm diameter disks were punched out from the thin foils and further electropolished down to electron transparency with a specific solution (1:2:9 perchloric acid, butoxyethanol, methanol) at −10 °C and 22 V, with a twin-jet polisher STRUERS TENUPOL-3 (Struers S.A.S., Milan, Italy). Phase identification was carried out through a Siemens D500 (Siemens, Munich, Germany), diffractometer equipped with Cu radiation tube with Ni filter on the tube side and monochromator on the detector side (0.05° step and 5 s counting time).

#### 2.5.4. Mechanical Tests

**Figure 4:**
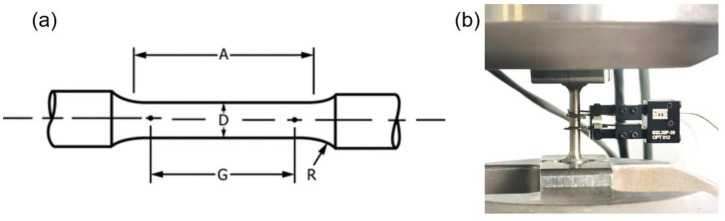
LPBF of Inc718; (**a**) tensile samples geometry, according to ASTM E8 (A = 20 mm, D = 4 mm, G = 16 mm, R = 4 mm), (**b**) tensile test set up.

Vickers microhardness measurements were performed on a Leitz™ DURIMET (Leica Microsystem S.r.l., Milan, Italy) testing machine using a load of 0.5 kg (i.e., a load of 4.905° N) with 30 s of dwell time. Ten measurements were performed in different areas of the polished surface of the sample. The uncertainty for microhardness measurement was calculated as the standard deviation on the measurement. Vertically oriented tensile test samples were directly manufactured according to ASTM E8 [22] as shown in Figure 4a using an alternating chessboard strategy with 3 mm × 3 mm rectangles and 90° of rotation between successive layers (Figure 2c). An MTS Minibionix servo-hydraulic testing machine (MTS System Corporation, Eden Prairie, MN, USA) having a load capacity of 15 kN equipped with an MTS TestStar IIm controller was adopted to perform tensile static tests on as-built, and heat-treated samples at room temperature. For each condition, three samples were tested for mechanical properties evaluation. The samples were loaded under displacement controlled at a rate equivalent to a strain rate of 10^−4^ s^−1^. The uniaxial MTS extensometer Model No. 632.29 F-30 having a gauge length of 5 mm was used to measure the strain as shown in Figure 4b.

## 3. Results and Discussion

### 3.1. Optimization of LPBF Process Parameters for Inc718

**Figure 5:**
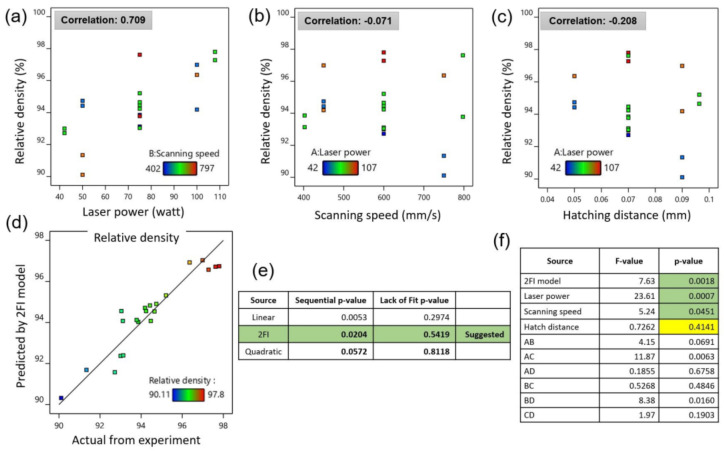
Optimization of micro-LPBF process parameters using response surface method for processing of Inc718; correlation between relative density and (**a**) laser power, (**b**) scanning speed and (**c**) hatch distance; (**d**–**f**) fitting a model corresponding to central composite design.

**Figure 6:**
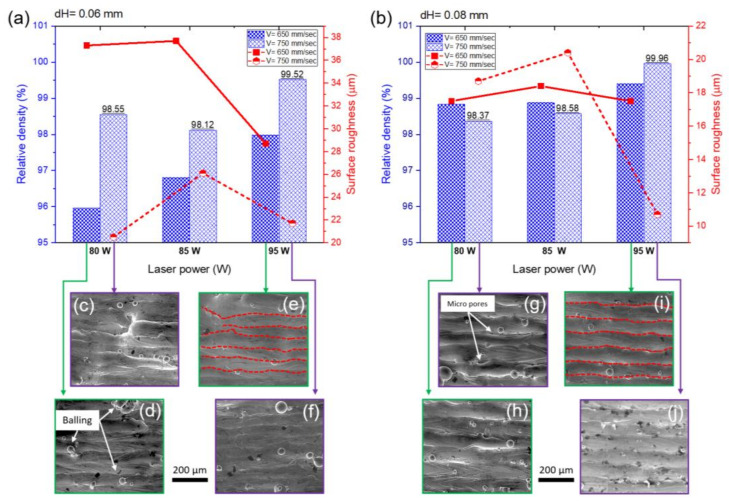
(**a**,**b**) Relative density and surface roughness of Inc718 samples produced by micro-LPBF; full factorial design with laser power of 80 W, 85 W, and 95 W, scanning speed of 650 mm/s, and 750 mm/s, hatch distance of 0.06 mm, and 0.08 mm; (**c**–**j**) surface topography of 12 samples from full factorial design showing surface defects such as porosity and balling.

The response surface method with a central composite design was employed to determine the optimum process parameter in LPBF of Inc718 using a spot laser of 30 µm. The relative density measurement of specimens produced by process parameters presented in Table 2 revealed a strong correlation between laser power and relative density (see Figure 5a). As can be seen in Figure 5b, a weak correlation was noted between scanning speed and relative density at this step. This can be explained by the fact that the maximum value of relative density in the first step was obtained to be lower than 98%. In the first DOE using RSM, relative density was considered as a single output that ranged from 90.11% to 97.8%. The model fit summary table corresponding to CCD is presented in Figure 5e. The 2FI model with a sequential *p*-value of 0.0204 was selected for further statistical analysis. Analysis of variance (ANOVA) for the selected model (2FI) showed a *p*-value of 0.0018 which confirms the significance of the selected model. The Model F-value was calculated to be 7.63 which implies that the model is significant. There is only a 0.18% chance that an F-value this large can occur due to noise. Details of the ANOVA are presented in Figure 5f for the selected model, input parameters, and model parameters; *p*-values less than 0.05 indicate model terms are significant. In this case, A, B, AC, and BD are significant model terms. In this model, A, B, C, and D factors represent laser power, scanning speed, hatch distance, and scanning strategy, respectively. Values greater than 0.1 indicate the model terms are not significant. The optimization module of the design expert software was used to predict the optimum process parameters aiming to achieve full density in LPBF Inc718 specimens. The optimization criteria were set on the maximum density and minimum surface roughness. In particular, five star and three star importance were considered for relative density and surface roughness, respectively. In order to develop the LPBF process for microfabrication, laser power of 95 W was considered a limit in the optimization process. Solution results of the optimization process showed that a set of process parameters consisting of laser power of 85 W, scanning speed of 737 mm/s, and hatch distance of 0.08 mm with an alternating bidirectional scanning strategy may lead to the full density LPBF product. This set of process parameters was then placed as the central point for the subsequent full factorial design of the experiment. Three levels of laser power (80 W, 85 W, and 95 W), two levels of scanning speed (650 mm/s and 750 mm/s), and two levels of hatch distance (0.06 and 0.08 mm) were considered in a full factorial design. Overall, relative density steadily increased up to the highest point above 99.9% by the raise in laser power, while it decreased by increasing scanning speed (Figure 6a,b). It is well-understood that excessive input energy density leads to evaporation-induced porosity [23]. As can be seen in Figure 6a,d, low scanning speed (650 mm/s) intensified evaporation and increased the porosity and balling effect. Comparing Figure 6a,b shows that the effect of scanning speed is more pronounced in samples produced by a lower hatch distance of 0.06 mm. Adjacent single tracks are highlighted by dashed red lines in Figure 6e,I which correspond to hatch distances of 0.06 mm and 0.08 mm, respectively.

It can be concluded that small hatch distances lead to distorted single tracks due to significant overlap between adjacent tracks. This phenomenon led to increased surface roughness in samples produced with 0.06 mm hatch distance (20–37 µm) in comparison with those with 0.08 mm hatch distance (10–18 µm).

From Figure 6a,b, it can be concluded that the sample produced by 95 W of laser power, 750 mm/s of scanning speed, and 0.08 mm of hatch distance presented the optimal condition due to its highest relative density (˃99.9%) and lowest surface roughness. This set of process parameters was then utilized for the fabrication of all samples that underwent the various heat treatment cycles.

### 3.2. Microhardness

**Figure 7:**
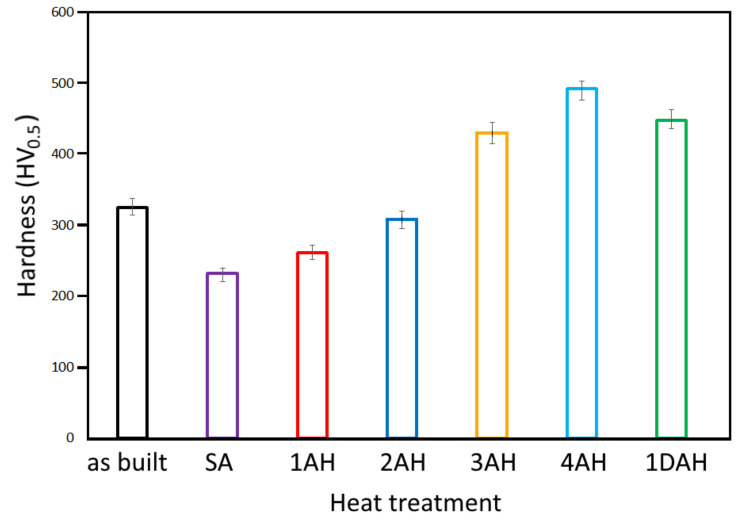
The average values of Vickers hardness in micro-LPBF Inc718 samples before and after different heat treatment cycles.

The microhardness measurement was performed on the samples produced by optimized parameters, to evaluate which heat treatment cycles induced the higher hardness values. The results are reported in Figure 7. The hardness of as-built samples resulted in 324 HV, slightly higher than reported values in the literature. For example, Tucho et al. [24] reported 288 ± 7 HV and 304 ± 9 HV for vertically and horizontally printed samples, respectively. Xing Li et al. [25] reported a hardness value of 300 HV for as-built Inc718. This difference can be attributed to a finer grain microstructure induced by small melt pool dimension and rapid solidification using the spot laser of 30 µm. As can be seen in Figure 7, hardness dropped from 324 HV to 232 HV after solution annealing. This loss of hardness can be attributed to the high temperature of solution annealing treatment (1080 °C) that induced a complete recrystallization. A single-step solution annealing at 620 °C for 4 h (1AH) and 8 h (2AH) did not increase the hardness significantly (261 HV and 308 HV, respectively). This can be easily attributed to low temperature and ageing treatment which led to the insufficient precipitation of secondary phases. Increasing the ageing temperature from 620 °C to 720 °C showed a substantial increase in hardness, and the values of 430 and 498 HV were reached after 4 h (3AH) and 8 h (4AH) of treatment, respectively. With double age hardening (1DAH), the hardness reached values of approximately 450 HV.

In conclusion, a single treatment for 8 h at 720 °C (4AH) resulted in the highest value (approximately 500 HV) of hardness. Double ageing treatment at 720 °C and 620 °C for 4 h (1DAH) showed the second-highest value of hardness (450 HV).

After this preliminary test, the results of samples 4AH and 1DAH were considered the most promising in terms of hardness value and time of treatment; therefore, the subsequent characterizations focused mainly on the comparison of these two samples with as-built and solution annealed samples.

### 3.3. Microstructure

#### 3.3.1. Micro-LPBF As-Built Inc718

**Figure 8:**
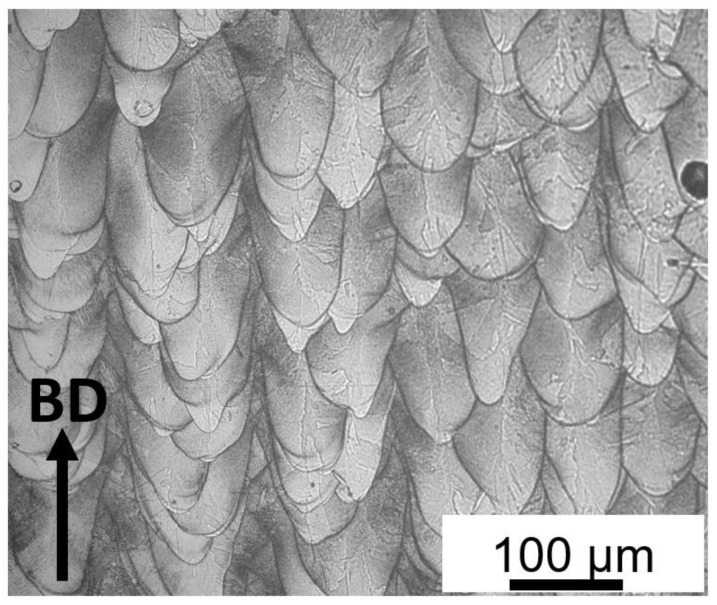
OM image of as-built micro-LPBF Inc718.

Observation of the as-built sample in the building direction using optical microscope showed a typical LPBF microstructure as presented in Figure 8. LPBF microstructure is characterized by melt pools and specific features such as columnar dendrites, columnar grains, and cellular sub-structures whose origin can be traced to the high thermal gradients of the process.

**Figure 9:**
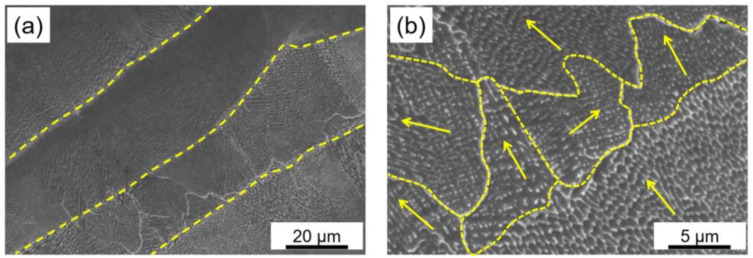
SEM micrographs of as-built sample parallel to the building direction; (**a**) SEM image in low magnification shows columnar/cellular structure in successive layers; (**b**) grains and columnar/cellular sub-grains (arrows in (**b**) indicate dendrite growth directions).

SEM analysis of as-built samples evidenced the presence of cellular and dendritic sub-structures (Figure 9a) parallel to the building direction. Similar microstructures are reported in the literature for Inc718 produced by LPBF [14,15]. As shown in Figure 9b, a fine microstructure was formed because of the high cooling rate induced by high input energy density (grain boundaries are indicated by yellow dash lines). As shown by the yellow arrows in Figure 9b, dendrite growth direction varies in different melt pools which indicates various temperature gradients in adjacent melt pools. It should be mentioned that the overall heat flow direction was parallel to the building direction [7]. However, the heat flow directions were not necessarily parallel to the building direction. It is well-understood that process parameters such as scanning strategies and thermal fields can significantly affect the heat flow directions and consequently dendrite growth direction [26]. Primary dendrite arm spacing was approximately 100–200 nm which suggests a very fine cellular-dendrite microstructure in comparison with the previous reports [27]. The grain size distribution was inhomogeneous with an average value of 10 μm. Each grain contained several columnar/cellular sub-grains.

**Figure 10:**
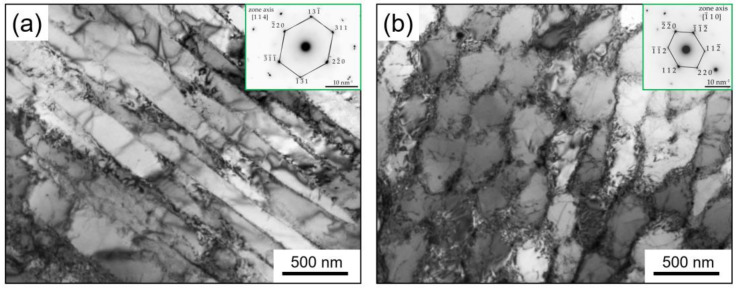
TEM bright field images showing (**a**) columnar and (**b**) equiaxed cellular substructure in as-built Inc718 sample produced by micro-LPBF. Inserts show the corresponding SAD patterns.

TEM analysis revealed more details of the columnar and cellular substructure of the as-built sample as presented in Figure 10a,b, respectively. Cellular substructures suggest equiaxed sub-grains whereas a columnar substructure consists of elongated sub-grains. In addition, the cellular microstructure was confirmed to be γ phase from the SAD pattern identified in Figure 10b. The average thickness of columnar sub-grains and average equiaxed sub-grains size was measured to be the same at 200 nm. A network of dislocations entangled at the low angle grain boundaries of cellular substructure can be seen in Figure 9b. The high-density dislocation microstructure of the as-built sample is due to the plastic deformation caused by residual stresses, induced in turn by the high thermal gradient and rapid solidification typically present in the LPBF process [13,28]. The same dimension for columnar and cellular sub-grains of 500 nm was previously reported by Tucho et al. [24]. Xing Li et al. reported 700 nm for the average dimension of cellular substructure in LPBF of Inc718 by using a laser spot diameter of 70 µm [25]. Moreover, the grain size distribution of 14.9 μm and a fine sub-structure of 452 nm were reported by Huang et al. [13] in LPBF of Inc718 using a self-developed LPBF machine equipped with a 500 W fiber laser. The finer columnar/cellular sub-grain structure in the current study with respect to the literature can be attributed to the smaller spot laser used in the micro-LPBF process (30 µm). A smaller spot laser diameter led to the formation of a smaller melt pool. The cooling rate of a small melt pool is very high which resulted in a steep temperature gradient. On the other hand, a steep temperature gradient is known as the main driving force for recrystallization and consequent fine microstructure of LPBF built parts [12,13].

#### 3.3.2. Solution Annealing of Inc718 Produced by LPBF

**Figure 11:**
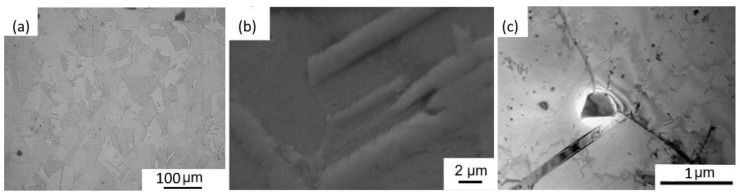
(**a**) OM image; (**b**) SEM image, and (**c**) TEM image of solution annealed micro-LPBF Inc718.

In this work, solution annealing at 1080 °C for 1.5 h was performed as the initial step in all heat treatment procedures, as presented in Table 4. OM, SEM, and TEM observations of the SA sample evidenced a notable change in the microstructure in comparison with the as-built samples: the melt pools disappeared, and recrystallization and grain growth occurred with the formation of grains ranging between 30 and 80 µm (Figure 11a). The presence of annealing twins was also detected (Figure 11a–c), which are typical in metals with an FCC structure subjected to annealing treatment [29].

#### 3.3.3. Ageing Hardening of Inc718 Produced by LPBF

**Figure 12:**
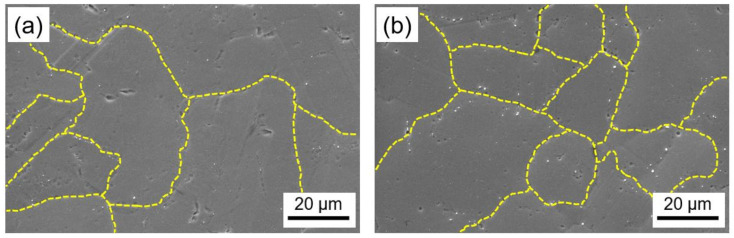
SEM image of (**a**) 4AH and (**b**) 1DAH samples of micro-LPBF Inc718.

Considering the results of the hardness test, samples 4AH (720 °C for 8 h) and 1DAH (720 °C for 4 h + 620 °C for 4 h) were selected for further microstructural analysis. The SEM images of low magnification evidenced the grain size of the two samples. The 4AH sample was characterized by a slightly larger grain size in comparison with the 1DAH one due to a longer holding at one higher temperature (Figure 12). To study the microstructure more in detail, observations were also performed with a Field Emission SEM.

**Figure 13:**
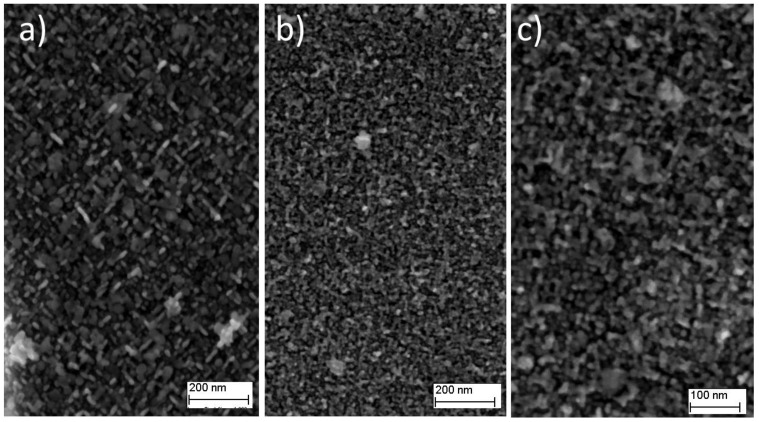
FESEM image of (**a**) 4AH and (**b**,**c**) 1DAH samples of micro-LPBF Inc718.

The microstructure of the two samples was characterized by the presence of very fine precipitates homogeneously distributed in the matrix. In the 4AH sample, it was possible to discern the spherical precipitates that suggested the presence of γ′ phase, and long disc-shaped ones, attributed to γ″ phase (Figure 13a). The size of both phases varies between 10 and 25 nm. In the 1DAH sample, the microstructure was smaller compared with the 4AH one (Figure 13b) and only at higher magnification it was possible to distinguish the presence of γ′ from γ″ (Figure 13c). The size of the phases resulted in approximately 10 nm. To deeply analyze the microstructure of 1DAH samples, TEM observations were carried out.

**Figure 14:**
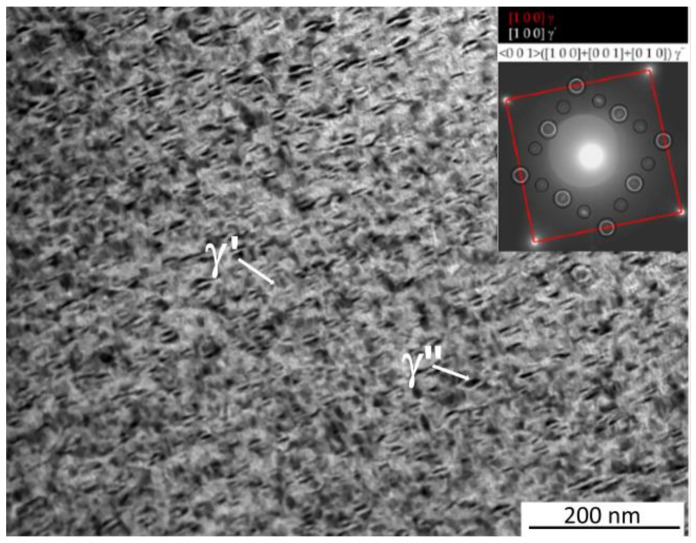
TEM bright field images of 1DAH sample with corresponding SAD patterns of γ and γ″ phases along the [$1\bar{1}2$] and [$1\bar{1}1$] zone axes, respectively.

Figure 14 shows the bright-field TEM image that revealed the presence of very fine (<10 nm) circular γ′ and plate-like γ″ particles distributed homogenously in the γ matrix. A typical SAD pattern with zone axis <001> is shown in Figure 14. It can be seen that all the reflections of the three types of orientation relationship of γ″ and reflection of the [010]γ″ are stretched along the [010]γ″ direction, while [100]γ″ are stretched along the [001]γ″ direction, no stretching of the [001]γ″ variant can be noted.

### 3.4. X-Ray Diffraction

**Figure 15:**
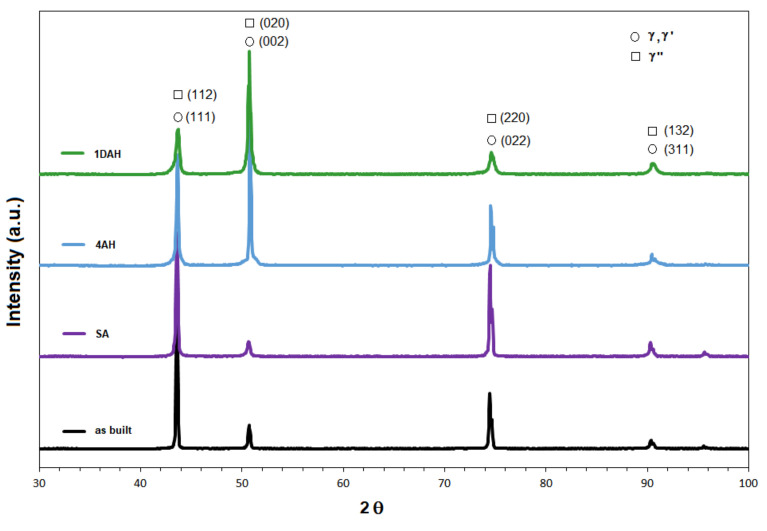
XRD patterns of the micro-LPBF Inc 718 samples before and after heat treatment.

The XRD patterns from the cross-sections of the as-built and heat-treated (SA, 4AH and 1DAH) samples in the building direction are shown in Figure 15. The main peaks observed for the as-built and SA samples were from the matrix, γ phase, and no peaks related to the presence of δ and the Laves phase were observed. The XRD patterns of 4AH and 1DAH samples are similar to ones of as-built and SA samples: the only difference is an enlargement of the peaks. The peaks of γ′ and γ″ phases cannot be distinguished since the lattice constants of γ, γ′, and γ″ phases are very close and each of the diffraction peaks was an overlap of peaks associated with the individual phases. The enlargement of the peaks in 4AH and 1DAH samples was most likely due to the presence of γ′ and γ″ phases. To separate the overlapping peaks and to calculate the volume fractions of γ, γ′, and γ″ phases, the Gaussian fitting method in the Origin software was used in the literature [24,30,31]. In this work, the Gaussian fitting method present in the HighScore Plus software was employed.

**Figure 16:**
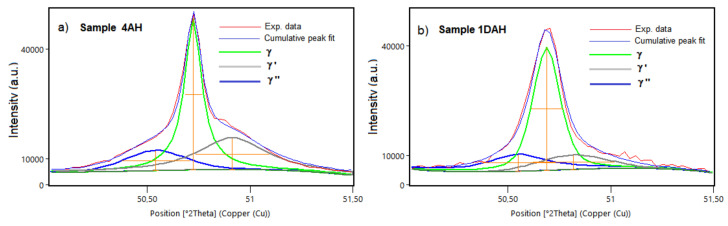
The fitting results of the peaks 200 for (**a**) 4AH sample and (**b**) 1DAH sample.

The fitting results on the strongest peak 002 in 4AH and 1DAH samples are shown in Figure 16. Based on the integral area of the separated peaks, the volume fractions of phases in heat-treated specimens were: 31% of γ′ and 16% of γ″ in the 4AH sample, 16% of γ′ and 20% of γ″ in the 1DAH sample. These results evidenced that the prolonged heat treatment induced a higher precipitation of γ′ phase than γ″, whereas the double treatment promoted the precipitation of γ″.

### 3.5. Mechanical Tests

#### 3.5.1. Uniaxial Tensile Test

**Figure 17:**
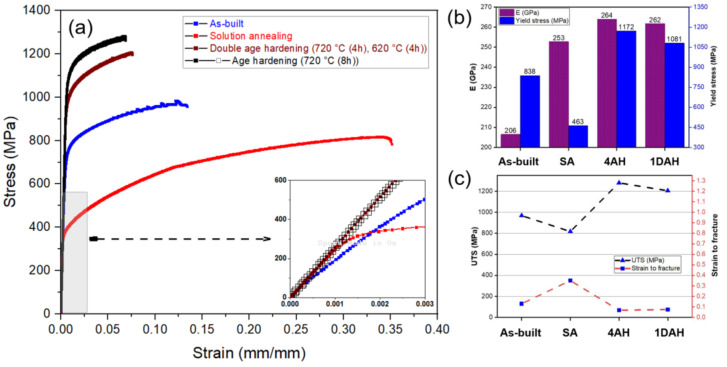
(**a**) Tensile stress-strain curves, (**b**) elastic modulus and yield stress, (**c**) ultimate tensile strength and strain to fracture Inc718 processed by micro-LPBF before and after heat treatment cycles.

Mechanical performance of as-built and heat-treated micro-LPBF Inc718 samples was investigated through a uniaxial tensile test at room temperature. The stress-strain curves are presented in Figure 17a. As can be seen in Figure 17a, both single age hardening (sample 4AH), and double age hardening (1DAH) heat treatments significantly increased the yield strength and ultimate tensile strength. Figure 17b shows 838 MPa for a yield stress of the as-built sample which is higher than reported values for LPBF of Inc718 using larger laser spot sizes (80–110 µm). Deng et al. [32] reported yield stresses of 780 MPa and 600 MPa for horizontally and vertically built Inconel 718, respectively. Popovich et al. [33] reported a yield stress of 668 MPa and 531 MPa for LPBF Inc718 samples using 250 W and 950 W of laser power, respectively. As mentioned earlier, a combination of small laser spot diameter (30 µm) and thin layer thickness generates a small melt pool and leads to a much higher cooling rate than those in larger laser spots. This phenomenon leads to a finer microstructure that increases the yield stress in samples produced by micro-LPBF.

As presented in Figure 17b, the 28% increase in yield stress and 39% increase in ultimate tensile strength were obtained after single-step age hardening. The high tensile strength of aged samples can be attributed to the formation of strengthening phases γ′ and γ″ during the ageing treatment as was previously shown using FESEM (Figure 13) and TEM (Figure 14). As presented in Figure 17c, age hardening substantially decreased the strain to fracture in both 4AH and 1DAH samples. Single-step age hardening showed the highest strength and the lowest ductility in comparison with the as-built, solution annealed, and double age hardened samples. This can be attributed to the fine and homogenously distributed precipitates (γ′ and γ″) within the γ matrix as presented in Figure 13 and Figure 14. Moreover, the XRD analysis revealed that the presence of γ′ in a single age hardening sample (4AH) is more pronounced compared with the 1DAH sample, hence higher strength of the 4AH sample was predictable. As can be seen in Figure 17a, as-built Inc718 samples showed higher tensile strength than the solution annealed sample. As mentioned earlier, rapid cooling and consequent severe thermal gradients lead to a large amount of residual stress in as-built samples [34]. This residual stress can cause plastic deformation that creates a high density of dislocations at grain boundaries as shown in Figure 10. After solution annealing and subsequent recrystallization, the as-built work hardened microstructure transforms into a stress-free microstructure with lower hardness and tensile strength. It is noteworthy to mention that the maximum strength of micro-LPBF samples after age hardening was comparable to those reported in the literature.

#### 3.5.2. Fractography

**Figure 18:**
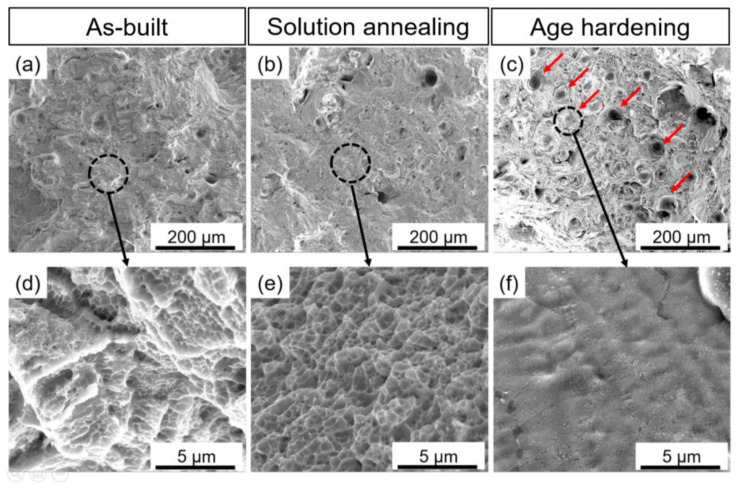
Fracture surfaces of micro-LPBF processed Inc718 samples after uniaxial tensile tests; (**a**,**d**) as-built sample, (**b**,**e**) solution annealed sample, (**c**,**f**) single-step age hardened sample at 720 °C for 8 h (4AH).

The SEM micrographs of the fracture surfaces of as-built, solution annealed, and 4AH samples after uniaxial tensile tests are presented in Figure 18. In the age hardening sample, the fracture surface contains dark circular regions which are shown by small red arrows in Figure 18c. These dark regions indicate the complete detachment of the material during the tensile test. Higher magnification of the fracture surface related to age hardening samples is presented in Figure 18f. Flat surfaces in this sample can be attributed to the brittle fracture behavior of age hardening samples which is in agreement with its low strain to failure value presented in Figure 17c. The fracture surface of solution annealing samples showed a homogenous presence of small dimples which is the characteristic of the dominant ductile fracture (Figure 18b,e). Nearly fully ductile fracture of the solution annealing sample led to the high value of strain to failure (35%) which is presented in Figure 17c. In addition, surface fractography of the as-built sample revealed a mixed ductile-brittle fracture mode by the presence of a mixture of flat surfaces and small dimples as presented in Figure 18a,d.

## 4. Conclusions

In this study, the laser powder bed fusion process of Inc718 with specific parameters developed for micro fabrication was examined. By employing a comprehensive design of the experiment, the optimization of main process parameters was carried out aiming to produce the full relative density samples with the minimum surface roughness (relative density outweighed surface roughness in priority). The influence of different heat treatment cycles on the microstructure and mechanical properties of micro-LPBF-fabricated Inc718 were studied using different techniques. The grain structure and precipitates were characterized via SEM, FESEM, TEM, and XRD. Mechanical characterization was conducted by hardness measurements and room temperature tensile testing. Conclusions can be drawn as follows:

- The outputs of DOE were used to plot process maps. Maximum relative density (99.9%) and relatively minimum surface roughness (<10 µm) were obtained using 95 W of laser powder, 750 mm/s of scanning speed, and hatch distance of 0.08 mm;
- The as-built microstructure features distinctive columnar and equiaxed cellular substructures with an average size of 200 nm which is finer than reported values in the literature. This was attributed to the small melt pool induced by the micro-LPBF process and the consequently high recrystallization rate which led to fine microstructure;
- Precipitation of very fine spherical γ′ phase and long disc-shaped γ″ phase was detected in Inc718 samples subjected to solution annealing and subsequent age hardening. The FESEM and TEM analysis revealed that double age hardening resulted in smaller precipitates (<10 nm) compared with a single-step age hardening;
- Age hardening heat treatment significantly increased the yield stress (28% after single age hardening) and ultimate tensile strength (39% after single age hardening) due to the formation and even distribution of the strengthening γ′ and γ″ phases;
- Single age hardening (720 °C for 8 h) induced the precipitation of a higher amount of γ′ precipitates and higher values of mechanical strength than double age hardening (720 °C and 620 °C for 4 h).

## Figures and Tables

**Table 1:** Chemical compositions of Inc718 powder.

El	Ni	Cr	Fe	Nb	Mo	Ti	Al	Co	Si	Mn	Cu	C	P	N	B
**wt%**	53.70	17.93	18.17	5.20	2.96	0.95	0.48	0.33	0.08	0.08	0.05	0.025	0.009	0.004	0.0025

**Table 2:** Input ranges of process parameter used in CCD.

Parameter	Numeric Factors					
	Unit	−α	Low	Central	High	+α
**Laser Power**	W	42	50	75	100	108
**Scanning speed**	mm/s	400	450	600	750	800
**Hatch distance**	mm	0.04	0.05	0.07	0.09	0.1
	**Categorical factor**					
	Level 1			Level 2		
**Scanning strategy**	Bidirectional			Alternating Bidirectional		

**Table 3:** Industrial standard heat treatment cycles for wrought and casting Inc718.

	Step 1	Step 2	Step 3
Wrought Inc718 [20]	SA: 980 °C (1 h), AC	DAH: 720 °C (8 h), FC at 55 °C/h to 620 °C (8 h), AC	
Casting Inc718 [21]	H: 1080 °C (1.5 h), AC	SA: 980 °C (1 h), AC	DAH: 720 °C (8 h), FC at 55 °C/h to 620 °C (8 h), AC

**SA**: solution annealing; **H**: homogenization; **DAH**: double age hardening; **AC**: air cooling; **FC**: furnace cooling.

**Table 4:** Heat treatment cycles performed on Inc718 micro-LPBF samples.

Solution Annealing	Cooling 1	Ageing 1	Ageing 2	Cooling 2	Type
1080 °C, 1.5 h	AC	--	--	--	SA
1080 °C, 1.5 h	AC	620 °C, 4 h	--	WQ	1AH
1080 °C, 1.5 h	AC	620 °C, 8 h	--	WQ	2AH
1080 °C, 1.5 h	AC	720 °C, 4 h	--	WQ	3AH
1080 °C, 1.5 h	AC	720 °C, 8 h	--	WQ	4AH
1080 °C, 1.5 h	AC	720 °C, 4 h	620 °C, 4 h	WQ	1DAH

SA: solution annealed; AH: age hardening; DAH: double age hardening: AC: air cooling: WQ: water quenched.

## Data Availability

The raw/processed data required to reproduce these findings is available upon the request.
